# Tacrolimus‐Related Hepatic Sinusoidal Obstruction Syndrome in a Case Receiving Deceased Liver Donor Liver Transplantation

**DOI:** 10.1002/kjm2.70057

**Published:** 2025-06-06

**Authors:** Yu‐Tsun Hsieh, Pier‐In Liang, Meng‐Hsuan Chiang

**Affiliations:** ^1^ Department of Medical Imaging Kaohsiung Medical University Hospital, Kaohsiung Medical University Kaohsiung Taiwan; ^2^ Department of Pathology Kaohsiung Medical University Hospital, Kaohsiung Medical University Kaohsiung Taiwan

Hepatic sinusoidal obstruction syndrome (HSOS), also called hepatic veno‐occlusive disease (HVOD), is a rare disease resulting from sinusoidal endothelial cell injury that develops a narrowed venous lumen with reduced sinusoidal venous outflow, resulting in painful hepatomegaly, ascites, weight gain, and edema [1]. HSOS can occur in post‐transplant patients, and the majority of research has been carried out in post‐hematopoietic stem‐cell transplantation (HSCT) patients related to preconditioning treatment [2]. A limited number of cases of HSOS have been reported after renal, lung, pancreatic, and liver transplantations [1]. Here, we report a case of HSOS occurring after liver transplantation, in which tacrolimus might have played a causative role.

A 66‐year‐old male patient with HCV‐related liver cirrhosis had received a deceased liver donor liver transplantation because of decompensated liver cirrhosis (Child‐Pugh score A with hypoalbuminemia, ascites, and recurrent EV bleeding). His post‐transplant immunosuppressive drugs included tacrolimus, mycophenolate mofetil, and prednisolone. Three months after the transplant, massive right pleural effusion was discovered using ultrasonography during general surgery outpatient department follow‐up while other associated symptoms included progressive extremities edema and intermittent dyspnea. Laboratory examination revealed an elevated tacrolimus level (19 ug/dl).

The dynamic abdominal computed tomography (CT) revealed hepatic congestion and poor enhancement of the three major hepatic veins (Figure 1a). To differentiate tacrolimus‐related HSOS from hepatic venous outflow stenosis, venous angiography was performed via the right hepatic vein, revealing no obvious anastomotic stenosis, while the venous pressure measurement showed no significant gradient between donor IVC and recipient IVC. The hepatic veins were patent without thrombi (Figure 1b). The pathology of the liver biopsy revealed increased stromal fibrosis of the hepatic vein and a mild sinusoidal dilatation in the zone 3 area (Figure 1c) while the portal area showed mild acute and chronic inflammation without significant bile duct or blood vessel damage. The overall findings indicated tacrolimus‐related HSOS.

**FIGURE 1:**
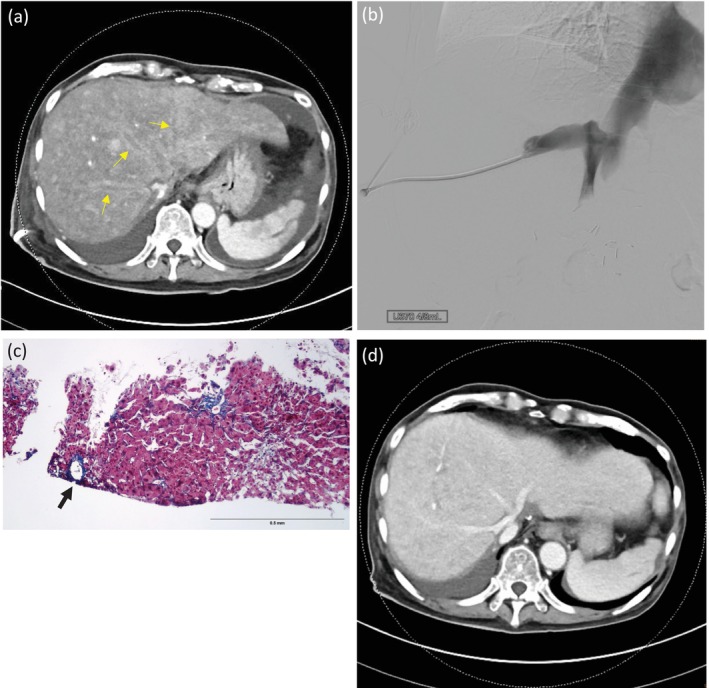
(a) The venous phase of the abdomen CT revealed poor enhancement of the major hepatic veins (arrows) and multiple small enhanced patches. (b) Venous angiography via the right hepatic vein showed patency of hepatic veins and IVC. (c) Masson's Trichrome stain (100x) showed perivenular fibrosis of hepatic veins (arrow). (d) Follow‐up dynamic abdominal CT revealed hepatic venous blood flow improvement.

After diagnosis, tacrolimus was replaced by cyclosporin. Two weeks after the management, a follow‐up dynamic abdominal CT revealed hepatic venous blood flow improvement (Figure 1d) and decreased ascites, with abdominal fullness being significantly alleviated. The patient was then discharged smoothly with cyclosporin, mycophenolate mofetil, and prednisolone as immunosuppressive medication.

HSOS is a rare complication in liver transplant recipients, with a reported incidence of 2% in the literature [3]. Although the incidence is low, HSOS can still cause graft failure. CT features of HSOS include patchy liver enhancement, main hepatic vein narrowing, and ascites [4], similar to our case and other HSOS cases [1, 4, 5], even if the cause is not tacrolimus [4]. The diagnostic gold standard relies on pathology, which is characterized by sinusoid congestion, fibrosis, and occlusion of hepatic lobular veins, and hepatic cell hemorrhagic necrosis [5].

To summarize, HSOS should be listed in differential diagnosis when CT findings mentioned above are presented in transplant recipients with tacrolimus administration. Tacrolimus is mainly metabolized via liver enzyme CYP3A4, so it should be used cautiously in liver‐impairment patients and in combination with medication metabolized by CYP3A4 (e.g., Amiodarone, Azole antifungal agents). Most importantly, monitoring tacrolimus level and adjusting accordingly is crucial to prevent tacrolimus‐related HSOS. Accurate diagnosis of HSOS and appropriate modification of medication can regain hepatic venous flow and improve associated symptoms.

## Conflicts of Interest

The authors declare no conflicts of interest.

## Data Availability

Data sharing is not applicable to this article as no new data were created or analyzed in this study.
